# An Episomal CRISPR/Cas12a System for Mediating Efficient Gene Editing

**DOI:** 10.3390/life11111262

**Published:** 2021-11-18

**Authors:** Nannan Duan, Shuqing Tang, Baitao Zeng, Zhiqing Hu, Qian Hu, Lingqian Wu, Miaojin Zhou, Desheng Liang

**Affiliations:** Center for Medical Genetics, School of Life Sciences, Central South University, Changsha 410078, China; duannnjj@163.com (N.D.); tangshuqing@sklmg.edu.cn (S.T.); zengbaitao2021@163.com (B.Z.); huzhiqing@sklmg.edu.cn (Z.H.); huqian@sklmg.edu.cn (Q.H.); wulingqian@sklmg.edu.cn (L.W.)

**Keywords:** Orip/EBNA1, CRISPR/Cas12a, gene editing, DMD, iPSCs

## Abstract

(1) Background: Gene editing technology, as represented by CRISPR is a powerful tool used in biomedical science. However, the editing efficiency of such technologies, especially in induced pluripotent stem cells (iPSCs) and other types of stem cells, is low which hinders its application in regenerative medicine; (2) Methods: A gene-editing system, COE, was designed and constructed based on CRISPR/Cas12a and Orip/EBNA1, and its editing efficiency was evaluated in human embryonic kidney 293T (HEK-293T) cells with flow cytometry and restriction fragment length polymorphism (RFLP) analysis. The COE was nucleofected into iPSCs, then, the editing efficiency was verified by a polymerase chain reaction and Sanger sequencing; (3) Results: With the extension of time, COE enables the generation of up to 90% insertion or deletion rates in HEK-293T cells. Furthermore, the deletion of a 2.5 kb fragment containing Exon 51 of the dystrophin gene (*DMD*) in iPSCs was achieved with high efficiency; out of 14 clones analyzed, 3 were positive. Additionally, the Exon 51-deleted iPSCs derived from cardiomyocytes had similar expression profiles to those of Duchenne muscular dystrophy (DMD) patient-specific iPSCs. Moreover, there was no residue of each component of the plasmid in the editing cells; (4) Conclusions: In this study, a novel, efficient, and safe gene-editing system, COE, was developed, providing a powerful tool for gene editing.

## 1. Introduction

Gene editing techniques, including zinc finger nucleases (ZFNs) [1,2], transcription activator-like effector nucleases (TALENs) [3,4,5], and clustered regularly interspaced short palindromic repeats (CRISPR) [6,7,8], have been harnessed as powerful tools for manipulating the genome in a myriad of organisms. In particular, the CRISPR and CRISPR-associated protein (Cas) system has redefined biomedical research due to its simplicity and ease of design. In this system, Cas proteins generate double-stranded breaks (DSBs) in specific sites. DSBs will then be repaired by the cell’s endogenous DNA repair machinery through non-homologous end-joining (NHEJ) or homology-directed repair (HDR), resulting in small insertions and deletions (indels) that have specific effects on gene expression [6].

The CRISPR/Cas9 system is the most commonly used Cas nuclease for recognizing GC-rich sites for restriction by protospacer adjacent motif (PAM), which greatly limits its application in non-coding regions enriched with AT [9]. In 2015, a new CRISPR gene-editing system, named CRISPR/Cas12a (also known as CRISPR/Cpf1), with a superior performance was found in *Prevotella* and *Francisella* 1 [10]. It can recognize T-rich PAM guided by a single CRISPR RNA (crRNA) and produces a double-stranded fracture with a sticky end. The system has expanded the application scope of gene editing and has been widely used in a variety of animals and plants [10,11,12]. In addition, CRISPR/Cas12a tends to have higher specificity than CRISPR/Cas9 [8,13,14,15,16].

A common problem associated with CRISPR/Cas9 or CRISPR/Cas12a is the low editing efficiency, especially in stem cells such as induced pluripotent stem cells (iPSCs) [10,17]. Additionally, various modes of CRISPR/Cas9 delivery have been investigated, including plasmids carrying the Cas9 gene [18], purified Cas9 mRNA [19], and ribonucleoprotein (RNP) [20]. However, plasmids are inefficient and RNP or mRNA purification is complicated and time-consuming. iPSCs hold great promise in regenerative medicine, drug screening, and biomedical research [21,22,23,24]. Therefore, a convenient and effective way to improve the CRISPR editing efficiency in iPSCs is urgently needed.

Orip/EBNA1, derived from the Epstein-Barr virus, has a post-synthesis function, which enables a plasmid containing Orip/EBNA1 to replicate autonomously in eukaryotic cells without integrating into the host cell genome [25,26]. Furthermore, it can be gradually lost during cell proliferation, as its replication cycle is slower than the cell division cycle [27]. Taking advantage of this feature, the episome gene-editing system, which combines CRISPR/Cas9 with Orip/EBNA1, has shown a high editing efficiency [28]. However, the potential usefulness of Cas12a with Orip/EBNA1 has yet to be demonstrated.

In this study, the effect of integrating CRISPR/Cas12a with Orip/EBNA1 (COE) on gene editing was investigated. It was found that the editing efficiency of the COE system increased with the culture time and enabled the efficient editing of endogenous genes in human embryonic kidney 293T (HEK-293T) cells and iPSCs, thus providing a good alternative for efficient gene editing.

## 2. Materials and Methods

### 2.1. Design and Construction of Episomal CRISPR/Cas12a

First, the AsCas12a element was cut with *Kpn*I (R3142, New England Biolabs, Ipswich, MA, USA) and *Xho*I (R0146S, New England Biolabs) from the pcDNA3.1-hAsCpf1 plasmid (Addgene #69982, MA, USA), and the U6 promoter was amplified from px330 vector (Addgene #42230,) with 2× ACE Taq Master Mix (Vazyme, Nanjing, China) on a Polymerase Chain Reaction (PCR) apparatus (Applied Biosystems, Foster City, CA, USA). The PCR reaction steps were as follows: 95 °C for 5 min, followed by 35 cycles of 95 °C for 30 s, 60 °C for 30 s, 72 °C for 30 s, and a final extension step at 72 °C for 10 min. Additionally, AsCas12a and the U6 promoter were successively cloned into the pCEP4 vector (Thermo Fisher, Waltham, MA, USA) with T4 ligase (M0569S, New England Biolabs) which contains the CMV promoter and the Orip/EBNA1 elements and can help obtain the COE plasmid (Figure S1). Plasmids were transformed into competent *Escherichia coli* DH5α (Tiangen, Beijing, China) for amplification [29]. Then, plasmid extraction was performed using the QIAGEN plasmid extraction kit (QIAGEN, Hilden, Germany). Next, the Orip/EBNA1 elements were excised with *Bbs*I (R3539S, New England Biolabs) to construct a non-additive system as a control named Cas12a. In addition, the px330 vector containing the Cas9 element was used as another control group named Cas9. The insertion of crRNA or single guide RNA (sgRNA) binding sequences into COE/Cas12a or Cas9-based vectors was performed as previously described [30]. Descriptions of the crRNA and sgRNA target sites are available in Table S1. For sgRNA design, the RGEN (http://www.rgenome.net/cas-designer/, accessed date 9 November 2021) was used. All primers were procured from Sangon Biotech (Shanghai, China).

### 2.2. Cell Culture and Maintenance

HEK-293T cells were purchased from ATCC (Manassas, VA, USA) and cultured on 6 cm plates (Corning Incorporated, Corning, NY, USA) using Dulbecco’s Modified Eagle’s Medium (DMEM, Thermo Fisher) supplemented with 10% fetal bovine serum (FBS, Thermo Fisher). iPSCs [31] and Duchenne muscular dystrophy (DMD) patient-specific iPSCs (DMD-iPSCs) [32] were previously constructed by our laboratory; all subjects gave their written informed consent. iPSCs were cultured on Matrigel (BD Biosciences, Franklin Lakes, NJ, USA) coated plates (Corning) using high glucose mTeSR-plus cell culture medium (STEMCELL Technologies, Vancouver, Canada) and subcultured every 6 days. All the cells were cultured at 37 °C and 5% CO_2_ in a cell culture incubator (Thermo Fisher) [33]. The results of subsequent experiments were based on the HEK-293T line and iPSCs lines and cardiomyocytes.

### 2.3. Genome Editing of HEK-293T with COE System

Stable Green Fluorescent Protein (GFP)-expressing HEK-293T (293T-GFP) cells were obtained by a lentivirus infection [34]. Before transfection, 293T-GFP were cultured in a 12-well plate (Corning) at a density of 3 × 10^5^ cells per well with high glucose DMEM cell culture medium for 24 h. When they reached 70–80% confluence, the cells were transfected with 1.5 μg of COE-GFP, Cas12a-GFP, or Cas9-GFP, respectively, according to the manufacturer’s instructions, using a Lipofectamine 2000 (Thermo Fisher). After transfection for 24 h, the medium was supplemented with 400 μg/mL of hygromycin B (Thermo Fisher) to enable drug screening, which lasted for 48 h. After transfection for 15 or 21 days, cells were collected and subjected to flow cytometry analysis as previously described [35] to determine the percentages of GFP-positive cells.

### 2.4. Restriction Fragment Length Polymorphism (RFLP) Analysis

To further analyze the ability of the COE to perform gene knockout in HEK-293T cells, crRNAs for targeting two loci (*NRL* and *HBB*), which included the *Pst*I and *Bsr*I restriction sites, were designed. Plasmid transfections were performed as previously described. After screening, genomic DNA (gDNA) was isolated on day 5, day 10, day 15, or day 20 using phenol-chloroform extraction [35]. The gDNA targeting sites were PCR-amplified as described for the first PCR. The purified PCR products were digested with restriction enzymes (*Pst*I and *Bsr*I) for 4 h and then agarose gel electrophoresis (180 V, 35 min) was performed using 1% agarose gel (Biowest, Barcelona, Spain). The gray value of each sample was generated via the ImageJ software (https://imagej.nih.gov/ij/index.html, accessed date 9 November 2021). All restriction enzymes were purchased from New England Biolabs.

### 2.5. Genome Editing of iPSCs with COE System

First, 1.6× 10^6^ of human iPSCs were disassociated and cultured in a 6 cm dish with mTeSR-plus medium for 24 h. When the cells reached 80% confluence, 8 μg of COE plasmid was used for nucleofection with the Amaxa Human Stem Cell Nucleofection Kit (Lonza, MD, USA). On day 3, cells were selected by hygromycin B for 72 h. For the analysis of single cell-derived clones, the cells were disassociated into single cells with TrypLE Express Enzyme without Phenol Red (Thermo Fisher) at day 10 post- nucleofection. They were then seeded onto the Matrigel-coated plates with mTeSR-plus medium for 15 days. Individual colonies were selected, and genotyped PCR was performed to screen for positive clones. The PCR primer’s sequences are shown in Table S1.

### 2.6. RNA Isolation and Quantitative Polymerase Chain Reaction (qPCR)

Total RNA was extracted from samples using Trizol (Thermo Fisher) extraction as previously described [36]. For this, 1 μg of RNA was used to reverse the transcription using the HiScript^®^ II Q RT SuperMix for qPCR (+gDNA wiper) Kit (Vazyme) according to the manufacturer’s instructions. qPCR was performed using the AceQ Universal SYBR qPCR Master Mix (Vazyme) with a CFX Connect RT-PCR detection system (Bio-Rad, Berkeley, CA, USA). The PCR primers sequence is shown in Table S1.

### 2.7. Cardiac Differentiation

The differentiation of iPSCs into cardiomyocytes was performed according to the method previously described [37]. Briefly, before differentiation, iPSCs were passaged at a 1:10 ratio, and cardiomyocytes differentiation was initiated when the cells reached about 80% confluence. During the differentiation, the medium was changed every other day. First, iPSCs were treated with CDM3 (a chemical-defined medium with three components) supplemented with CHIR99021 (Selleck, Shanghai, China) for 2 days. The CDM3 included recombinant human albumin (Sciencell Research Laboratories, Carlsbad, CA, USA), L-ascorbic acid 2-phosphate (Wako Chemicals, Osaka, Japan), and Roswell Park Memorial Institute (RPMI) 1640 basal medium (Thermo Fisher). Second, cells were cultured for another 2 days in CDM3 supplemented with WNT-C59 (Selleck). The medium was then replaced with the RPMI 1640 basal medium supplemented with a B27-supplement (Thermo Fisher) for 6 days, and the metabolic purification of the iPSC-CMs was carried out by replacing the medium with a RPMI 1640 medium with no glucose (Thermo Fisher), supplemented with a B27-supplement for 10 days. After being maintained in the RPMI 1640 basal medium supplemented with a B27-supplement for 6 days, the cardiomyocytes were dissociated with the STEMdiff™ Cardiomyocyte Dissociation Kit (STEMCELL Technologies).

### 2.8. Immunofluorescence Staining

Cells seeded on 24-well coverslips were fixed with cold acetone (Sigma-Aldrich, St. Louis, MO, USA) for 10 min followed by blocking with 5% bovine serum albumin (BSA, Sigma-Aldrich) for 30 min. After washing them three times with Dulbecco’s Phosphate Buffered Saline (DPBS, Thermo Fisher), the cells were incubated overnight with primary antibodies at 4℃. After incubation, the antibody solution was discarded, and the sections were each washed three times at room temperature for 5 min with DPBS. Next, the cells were blocked again followed by 1 h of incubation at room temperature with appropriate secondary antibodies. Nuclei were counter-stained with SSEA-1. 4′,6′-diamidino-2-phenylindole (DAPI, Selleck), and 24-well chamber slides were inverted onto glass slides overlaid with 90% glycerol (Sigma-Aldrich) in DPBS as the mounting media. Additionally, the last images were examined and captured using a confocal microscope (Leica DM IRB, Wetzlar, Germany).

The primary antibodies used were mouse anti-dystrophin (Developmental Studies Hybridoma Bank, Iowa City, IA, USA), anti-cardiac (Abcam, Cambridge, United Kingdom), Nanog (Abcam), tumor-related antigen (TRA)-1-60, TRA-1-81, stage-specific embryonic antigen (SSEA)-1, and SSEA-4 all from Merck Millipore (Darmstadt, Germany).

### 2.9. Residue of Plasmid Components and Off-Target Analysis

After genome editing, the iPSCs genomic DNA was extracted on days 40, 42, 48, 54, 60, 66, 72, and 75 using phenol-chloroform extraction, respectively. The residue of the plasmid components was tested by PCR. Additionally, the potential off-target sites predicted by an online tool (http://www.rgenome.net/cas-offinder/, last assessed on 25 September 2021) were amplified by PCR and Sanger sequencing. All primers are shown in Table S1.

### 2.10. Statistical Analysis

All the data are shown as the mean ± S.D. Statistical analyses were conducted using GraphPad Prism 8. Two-tailed, paired Student’s t-tests were used to determine statistical significance when comparing the two groups. A value of *p* < 0.05 was considered statistically significant.

## 3. Results

### 3.1. Establishment of the Episomal CRISPR/Cas12a System

**Figure 1:**
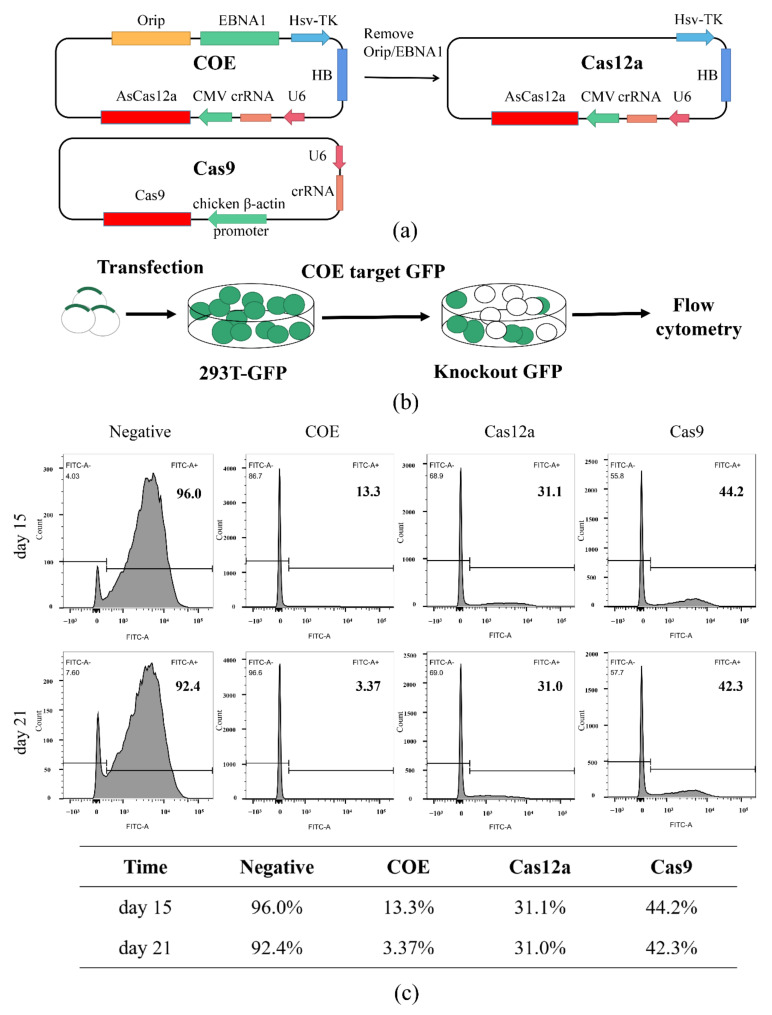
Cas12a-OriP/EBNA1 (COE) promotes gene editing GFP in HEK-293T. (**a**) Schematic of the COE, Cas12a, and Cas9 control system design. The COE contains U6 promoter (U6), CRISPR RNA (crRNA), CMV promoter (CMV), Cas12a, Hygromycin B (HB), and OriP/EBNA1 elements. The Cas12a system was designed by removing the OriP/EBNA1 elements of COE. (**b**) Schematic of genome editing in 293T-GFP. (**c**) Flow results of the GFP positive rates of cells on 15 and 21 days after transfection. On day 15, the GFP-positive rates of cells of COE, Cas12a, and Cas9 were 13.3%, 31.1%, and 44.2%, respectively; on day 21, these rates were 3.37%, 31.0%, and 42.3%, respectively. The data are shown in the table below.

An all-in-one Orip/EBNA1-based vector was constructed to express crRNA and AsCas12a, and the Orip/EBNA1 elements were excised to construct the control vector, non-Orip/EBNA1-Cas12a, which is named Cas12a (Figure 1a).

First, to assess the COE editing efficiency, a reporter cell line, 293T-GFP, was established via infection with a lentivirus containing GFP. The reporter-infected cells can stably express the GFP protein (Figure S2) while performing targeted editing on the open reading frame (ORF) of GFP. This consequently disrupts the GFP expression, which allows for the measurement of the editing efficiency through the monitoring of the GFP level using flow cytometry. crRNAs or sgRNAs were designed and constructed to target the ORF of GFP (Figure S3). Then, the COE, Cas12a, and Cas9 systems targeting GFP were transfected into 293T-GFP cells. The whole experimental process is shown in Figure 1b. We observed a decreasing rate of GFP-positive cells over time. On the 15th and 21st days after transfection, cells were collected and subjected to flow cytometry. The results showed that GFP-positive cells in the COE group were 13.3% on day 15 and 3.37% on day 21, respectively. Meanwhile, in the Cas12a and Cas9 groups, the rate of GFP-positive cells decreased to 31.1% and 44.2% on day 15. Moreover, the rate of positive cells did not decrease further during the detection period (Figure 1c). These results demonstrated that the COE system has a higher editing efficiency than Cas12a and Cas9, as this advantage became more obvious with the increase in cultivation time.

### 3.2. The COE System Significantly Promotes Gene Editing in HEK-293T

**Figure 2:**
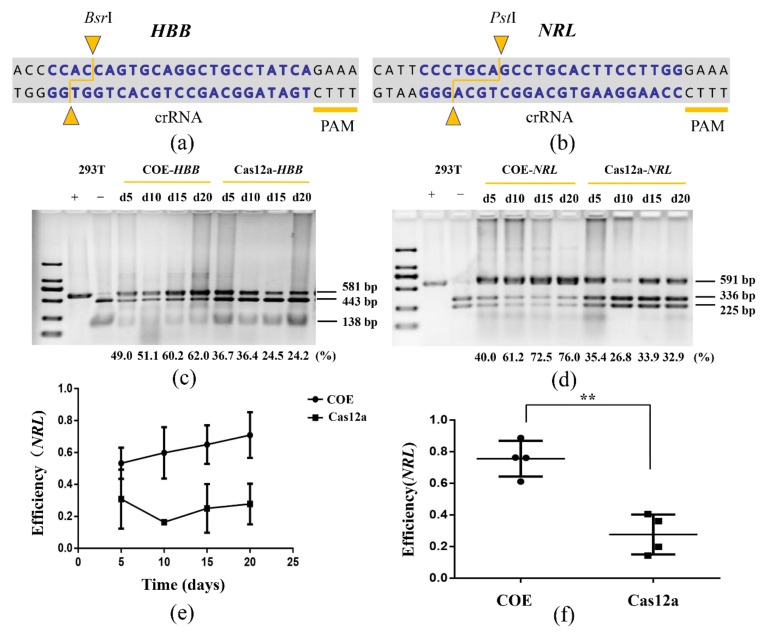
COE promotes gene editing in HEK-293T. (**a**,**b**) Schematic of *HBB* and *NRL* loci. Blue represents crRNA, yellow triangles represent the restriction enzyme cutting sites of *Bsr*I and *Pst*I. (**c**,**d**) RFLP analysis of COE and Cas12a cleavage at *HBB* and *NRL* sites. The sequence lengths were 581-base pair (bp) (*HBB*) and 591 bp (*NRL*). After enzyme digestion, there were two bands with sizes of 138 bp and 443 bp for the *HBB* site, and 336 bp and 255 bp for the *NRL* site. 293T+: without restriction enzyme-treated, 293T−: treated with restriction enzymes; d: day. The editing efficiency at each time point is shown in the figure with the digit. (**e**) RFLP analysis of the indel rates generated by the COE and Cas12a targeting *NRL* (*n* = 3). (**f**) Statistics for the editing efficiency of the *NRL* site on day 20 (*n* = 4, ** *p* < 0.01).

To further analyze the capacity of the COE system for gene editing endogenous loci of genomes, two crRNAs targeting two loci (*NRL* and *HBB*) located on different chromosomes of HEK-293T were designed (Figure 2a,b). These targeted loci contain restriction sites, which allowed us to design a simple polymerase chain reaction-restriction fragment length polymorphism (PCR-RFLP) assay to evaluate the modification efficiency. Increasing indel rates were observed over time. Twenty days after transfection, the editing efficiency of the *HBB* site increased from 49% to 62% and that of the *NRL* site increased from 40% to 76%. Meanwhile, no significant change was seen in the control group (Figure 2c–e). The editing efficiency of the COE group was three times that of the control group at the *NRL* site (Figure 2f). These results indicate that the COE system can be used as an efficient gene-editing tool in HEK-293T cell lines.

### 3.3. The COE System Efficient for Gene Deletion in Human iPSCs

**Figure 3:**
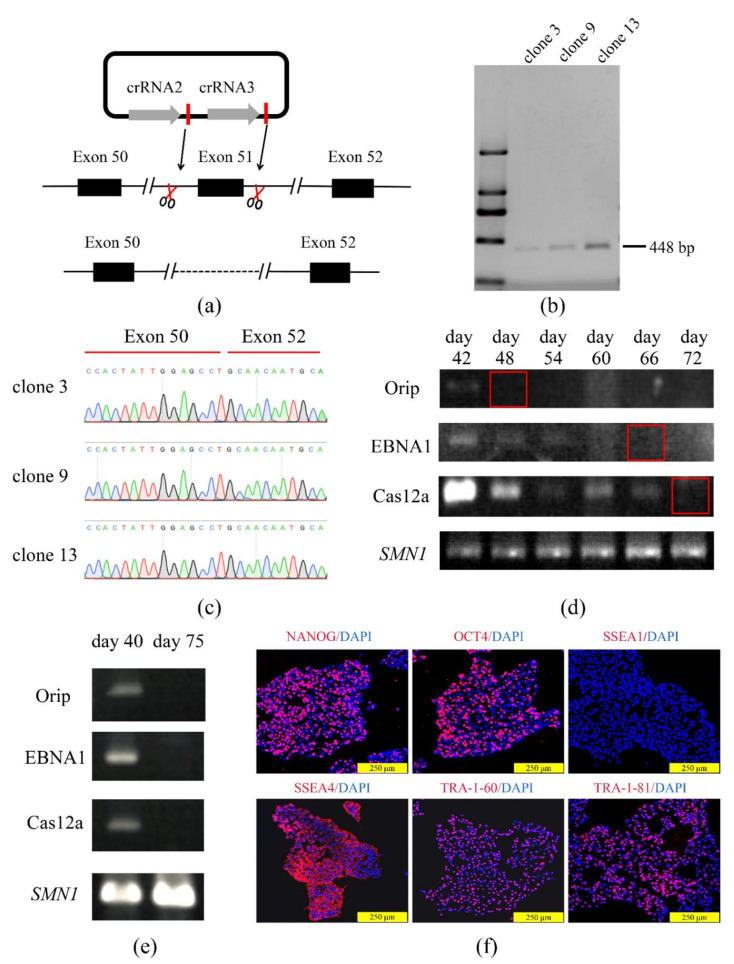
The COE system was found to be efficient for gene deletion in hiPSCs. (**a**) Schematic diagram of *DMD*-51 Exon targeting deletion. crRNA2 and crRNA3 were designed upstream and downstream of Exon 51. (**b**) PCR screening was performed for the clones using the primers F/R and the targeted deletion clones; clone 3, clone 9, and clone 13 had bands with the size of 448 bp. (**c**) Sequencing results showed that the Exon 51 was deleted in clone 3, clone 9, and clone 13. (**d**) Polymerase chain reaction (PCR) analysis of episomal DNA of transfected iPSCs on different days. Survival motor neuron gene 1 (*SMN1)* was used as internal reference. (**e**) Detection results of the plasmid residual of clone 3 on day 40 and day 75 after nucleofection. *SMN1* was used as an internal reference. (**f**) Immunostaining of Exon 51-deleted-iPSCs (D-iPSCs) markers for Nanog, Oct 4, SSEA-4, TRA-1-60, TRA-1-81, and differentiating marker, DAPI was used to visualize the nucleus. Scale bar, 250 μm.

DMD, a lethal X-linked recessive muscle dystrophy, is caused by different mutations including frame-shifting gross deletions and duplications of the *DMD* gene. About 13% of patients with DMD were found to have an Exon 51 deletion in a clinical study [38]. To determine the capacity of the COE system for gene knockout in hiPSCs as well as to construct a DMD cell model with an Exon 51 deletion, a two-cut crRNAs expression vector that can express two crRNAs for the targeted knockout of Exon 51 in hiPSCs was constructed (Figure 3a). The hiPSCs were transfected using the two-cut COE. Then, 14 mono-clones were picked up, and the Exon 51-deleted clones were identified and detected using PCR amplification with the primers F/R and confirmed by Sanger sequencing. Of the 14 clones found, the PCR and Sanger sequencing demonstrated that 3 Exon 51-deleted clones were obtained (Figure 3b, c, Figure S4). Additionally, one Exon 51-deleted clone (D-iPSCs) was randomly selected for further analysis. Since the episomal vector contains Orip/EBNA1 elements that can replicate autonomously in eukaryotic cells, it was determined whether the exogenous vector residual was retained in the Exon 51-deleted clone. The results showed that in the mixed cells without drug screening, no vector residuals were detected 72 days after nucleofection (Figure 3d, Figure S5). Additionally, in the D-iPSCs, no vector was detected on day 75 (Figure 3e, Figure S6). This is consistent with the finding of the previous report [39]. Next, a panel of potential off-target sites was analyzed using Sanger sequencing for crRNAs, these potential off-target sites were identified using an online tool (http://www.rgenome.net/cas-offinder/, last assessed on 25 September 2021), and the result revealed that no genome edition had taken place in any of the seven most probable off-target sites (Figure S7).

Further analysis revealed that the modified iPSCs displayed normal morphology and karyotype, while modified iPSCs maintained their pluripotent state, as indicated by the expression of pluripotency markers (Figure 3f).

### 3.4. The Edited iPSCs Were Directed to Differentiate into Cardiomyocytes

**Figure 4:**
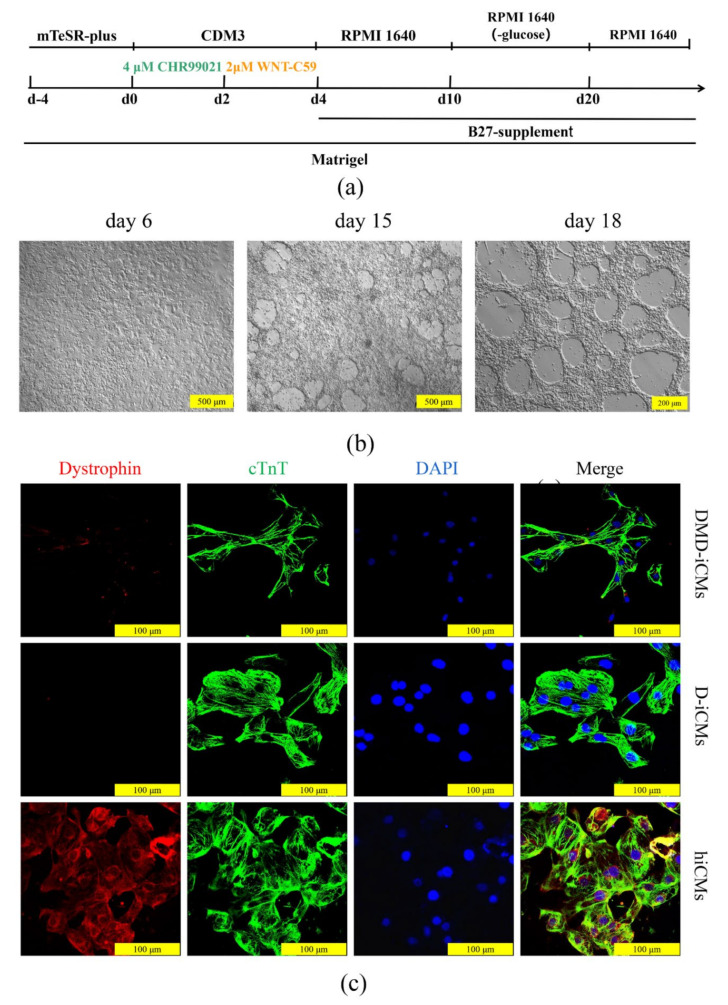
Differentiation of iPSCs into cardiomyocytes and dystrophin expression in cardiomyocytes. (**a**) Schematic protocol for cardiomyocytes differentiation. CDM3: chemical defined medium 3. RPMI 1640: Roswell Park Memorial Institute 1640. (**b**) Dynamic changes in cellular morphology during the differentiation of D-iPSCs-derived cardiomyocytes (D-iCMs). Scale bars, 500 μm and 200 μm. (**c**) Immunostaining of the cardiomyocyte-specific marker cardiac troponin T (cTnT) (green) and dystrophin (red) in iCMs. DAPI (blue) was used to visualize the nucleus. Scale bar, 100 μm.

The differentiation of iPSCs into cardiomyocytes was performed before and after Exon 51 was deleted, and the expression of dystrophin protein was evaluated in the constructed cell model. Cardiomyocytes from normal control hiPSCs (hiCMs), D-iPSCs (D-iCMs), and patient control DMD-iPSCs (DMD-iCMs) were differentiated by small molecules. The differentiation process is shown in Figure 4a. After 10 days under differentiation conditions, spontaneous contractions in cells could be observed (Figure 4b). After another 8 days, D-iPSCs, hiPSCs, and DMD-iPSCs were successfully differentiated into cardiomyocytes (Figure 4b and Figure S8). After purification, all iCMs were found to express the cardiomyocyte-specific marker cardiac troponin (cTnT). Additionally, the rare staining of the dystrophin protein was present in the DMD-iCMs and D-iCMs, and profusely in hiCMs (Figure 4c). These results indicate that the Exon 51 deleted cell model has a similar expression profile to that of DMD.

## 4. Discussion

In this study, an episomal CRISPR/Cas12a gene-editing system was established based on Orip/EBNA1 (COE) and its editing efficiency in HEK-293T and iPSCs was evaluated. Under the selection pressure, the editing efficiency of this COE system in HEK-293T cells was found to increase with culture time and could reach 90%. In theory, if the time is long enough, the COE system can achieve a higher indel rate. Additionally, the DMD model of the deletion of Exon 51 was successfully constructed using this system, which proved that this system can edit efficiently in iPSCs. At present, the most common method of gene knockout in iPSCs is to produce double-strand breaks (DSB) using programmable artificial endonucleases such as ZFN, TALEN, and Cas9, but the editing efficiency was very low. In 2019, Yi-Li Min et al. successfully knocked out Exon *DMD*-45 in iPSCs using CRISPR/Cas9, achieving a knockout efficiency of only 8% [40]. Additionally, Courtney S. Young successfully carried out the CRISPR/Cas9-mediated deletion of Exon 44–55 of *DMD* with an efficiency lower than 5% [38]. These efficiency rates mean that it is laborious and time-consuming to obtain homozygous knockout iPSC clones. In this study, the COE system successfully achieved the deletion of a 2.5 kb fragment containing *DMD* Exon 51 with high efficiency. Of the 14 clones analyzed, 3 were positive, demonstrating that the COE system is efficient and convenient. It is worth mentioning that the episomal vector can be removed after genome editing, eliminating the influence of exogenous genes on the results.

Compared with Cas9, Cas12a recognizes A-T rich sequences, where the PAM, typically 5′-TTTV-3′, fills the gap of gene editing in genomes rich in A-T sequences. Additionally, Cas12a presents a more minimalistic system than Cas9, as Cas12a requires only a single RNA molecule, the crRNA. Meanwhile, Cas9 requires two RNA molecules (tracrRNA and a crRNA). Previous studies also showed that the pre-crRNA processing activity of Cas12a makes it an attractive candidate for multi-gene editing, which is cumbersome with Cas9. Due to these characteristics, Cas12a is becoming another powerful gene-editing tool, playing an increasingly important role in nucleic acid detection, disease modeling, gene therapy, etc. In this study, its editing efficiency was significantly improved by the use of the Orip/EBNA1 episome, which undoubtedly provides another boost for its better use in scientific research. However, the episome derived from the Epstein-Barr virus (EBVs) also has some limitations. It has been reported that the EBV vector failed to replicate in mouse and hamster cells due to Orip, which may limit its applications for gene therapy trials in animals [41]. Therefore, some elements that mediate effective replication in organisms need to be inserted into the EBV vector to expand its application range.

In general, this gene-editing system provides a powerful platform for efficient genome editing. The COE system promotes genome editing in two ways: by allowing the continuous selection of transfected cells and long-term genome editing. The results of this study show that the editing efficiency increases over time; in theory, most crRNAs can generate indel rates of up to ~100% if the editing time is long enough. However, the sustained expression of Cas12a has created some risks as well as improved the editing efficiency. Some studies have shown that the CRISPR system can produce off-target cleavage [42,43], which greatly limits the use of the CRISPR system. Meanwhile, some studies have shown that the off-target effect is extremely low in iPSCs [44,45]. Compared with Cas9, Cas12a is more sensitive to mismatches in the guide RNA and has lower off-target effects [46]. In this study, no off-target cleavage was observed at the seven putative sites, even when the COE expression lasted 75 days. Therefore, the potential off-target effects are not a matter of concern for COE applications in disease modeling and other applications. Instead, its efficiency has greatly reduced the workload of researchers.

At present, nucleases can be delivered in the forms of DNA, mRNA, and protein, each with its advantages and disadvantages [5]. The protein delivery of nucleases is the most efficient because transcription and translation are avoided. Transient expression leads to fewer off-target effects [47]. However, the complicated protein purification process used increases the risk of contamination with bacterial endotoxin [48], immune responses [49], and inefficient delivery due to its charge and large size [50], which further limits its application. The mRNA-mediated delivery of nucleases is another system with great potential for clinical application. It initiates genome editing quickly since it does not need to be transferred into the cell nucleus [51] and provides better control over nuclease dosage and fewer off-target effects. However, mRNA is much less stable, the short half-life of mRNA limits its use greatly [52]. In practical applications, the delivery of mRNA is usually mediated through various non-viral synthetic materials [50], but cellular uptake, cytotoxicity, transfection efficiency, and other issues need to be considered. The DNA delivery of programmable nucleases is cost-effective and thus is of great value in basic research. In in vitro gene editing, a plasmid is still one of the most common vectors. In general, despite the rapid development of multiple delivery methods, the COE system still has high application value in disease modeling and other applications as it is efficient, cost-effective, and convenient.

## 5. Conclusions

In this work, an episomal CRISPR/Cas12a gene-editing system was established based on Orip-EBNA1. This system can efficiently implement gene editing in the HEK-293T and iPSCs, providing a powerful tool for efficient gene editing.

## Data Availability

All data supporting the reported result in this study can be found in the Supplementary Materials.

## Supplementary Materials

The following are available online at https://www.mdpi.com/article/10.3390/life11111262/s1. Figure S1: Schematic diagram of design and construction of episomal CRISPR/Cas12a. Figure S2: Stable GFP-expressing HEK-293T cell. Figure S3: Vectors targeting GFP were sequenced by Sanger sequencing. Figure S4: Sanger sequencing results of positive clones. Figure S5: Agarose gel electrophoresis diagram showing all bands and molecular weight markers of Figure 3d. Figure S6. Agarose gel electrophoresis diagram showing all bands and molecular weight markers of Figure 3e. Figure S7: Sequencing results of off-target sites. Figure S8: The morphology of cardiomyocytes. Table S1: All of the primers used in the study.
