# Supplementary material for: An Episomal CRISPR/Cas12a System for Mediating Efficient Gene Editing
Source: Life (Basel). 2021 Nov 18;11(11):1262. doi: 10.3390/life11111262 (PMC8620414; doi:10.3390/life11111262)
Supplement: Supplementary file 1 [file life-11-01262-s001.zip › life-1395813-supplementary.pdf]

life

Supplementary Materials

An Episomal CRISPR/Cpf1 System Mediated Efficient Gene Editing

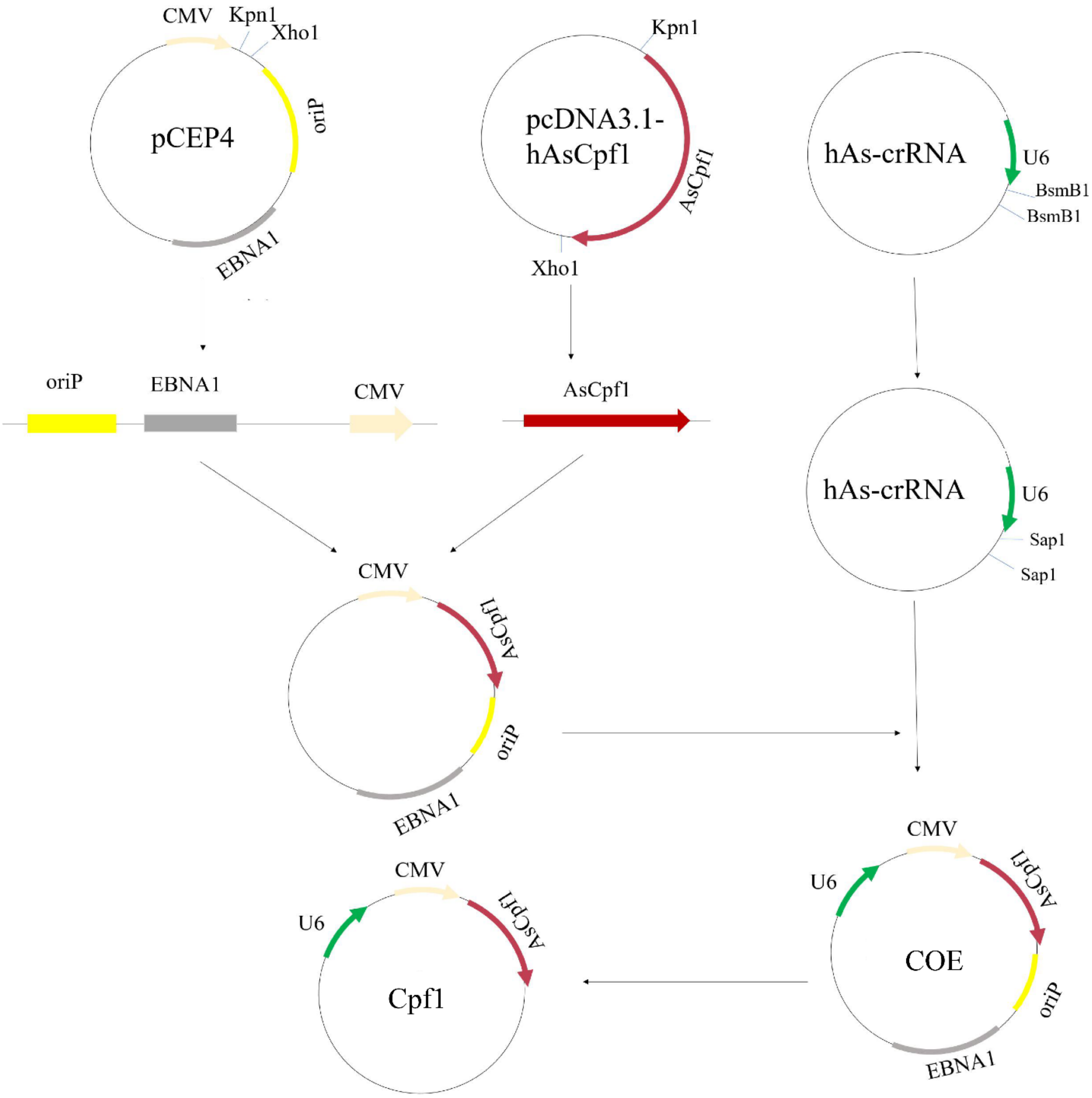

Figure S1. Schematic diagram of design and construction of episomal CRISPR/Cas12a.

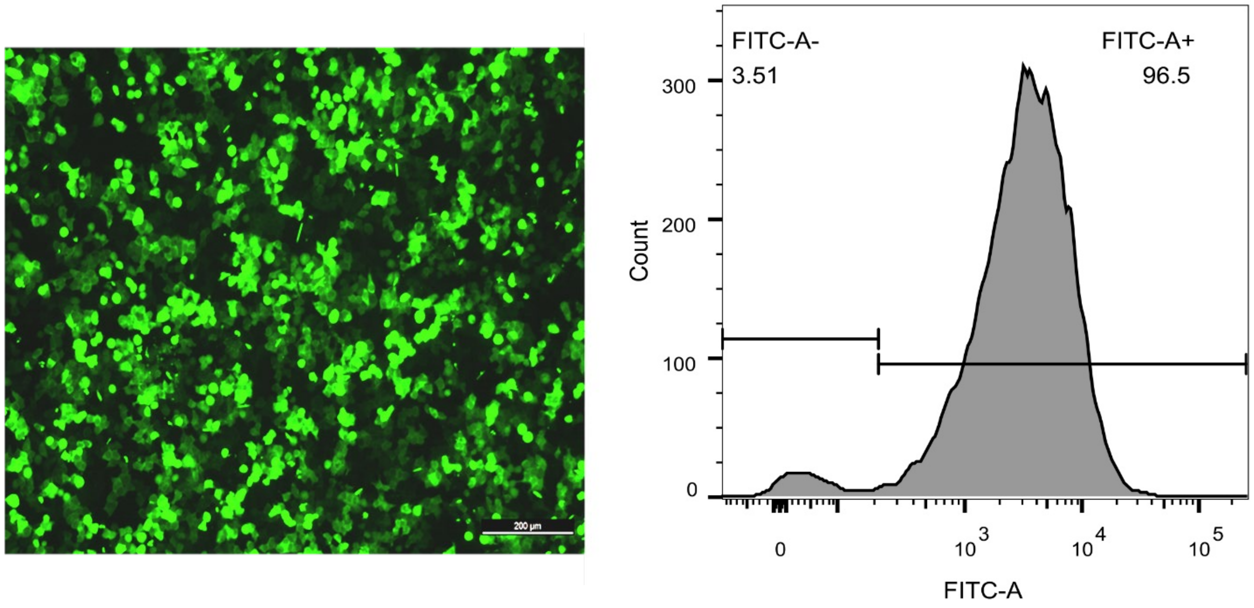

(a) **(b)**

Figure S2. Stable GFP-expressing HEK-293T cell. (**a**) Image of fluorescence signal of HEK-293T stably expressing GFP. Scale bar: 500 µm. (**b**) Flow results show that the rate of GFP positive cells was 96.5%.

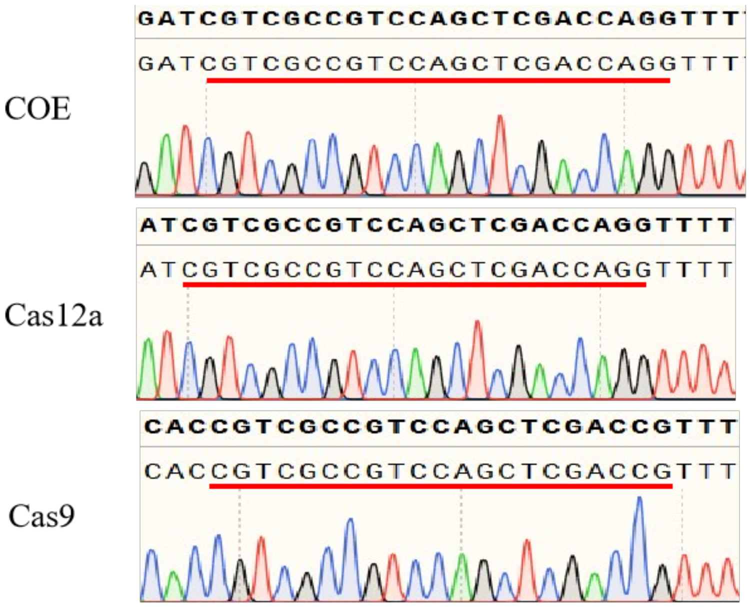

Figure S3. Vectors targeting GFP were sequenced by Sanger sequencing. Sanger sequencing results showed that vectors targeting GFP was successfully construct.

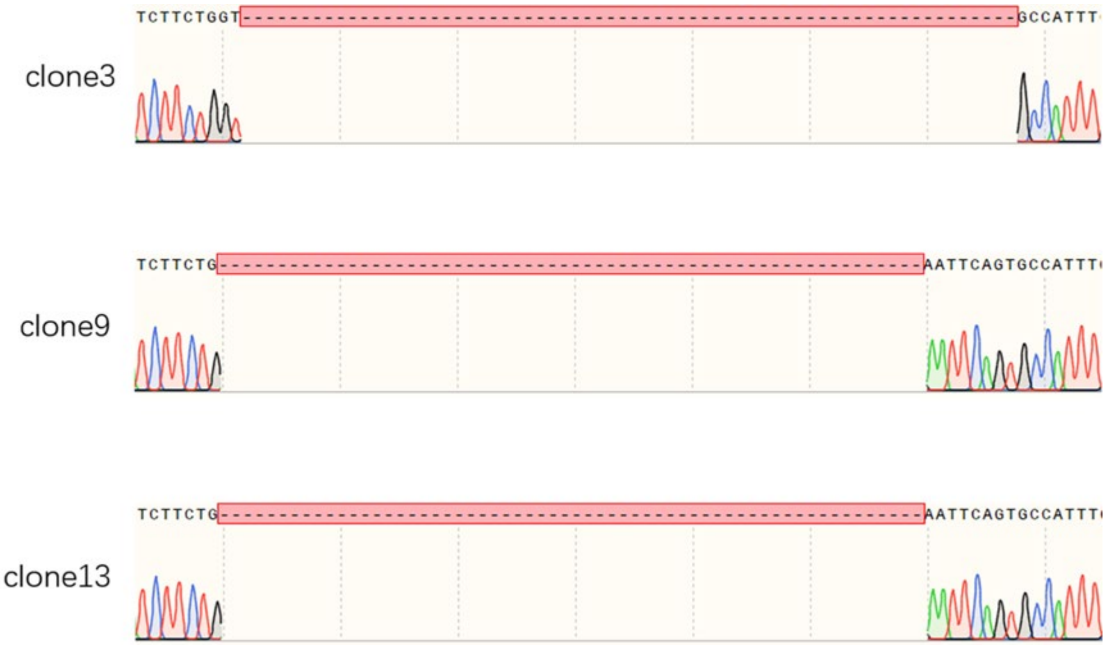

Figure S4. Sanger sequencing results of positive clones. Sanger sequencing results showed that Exon 51 was successfully deleted from three positive clones.

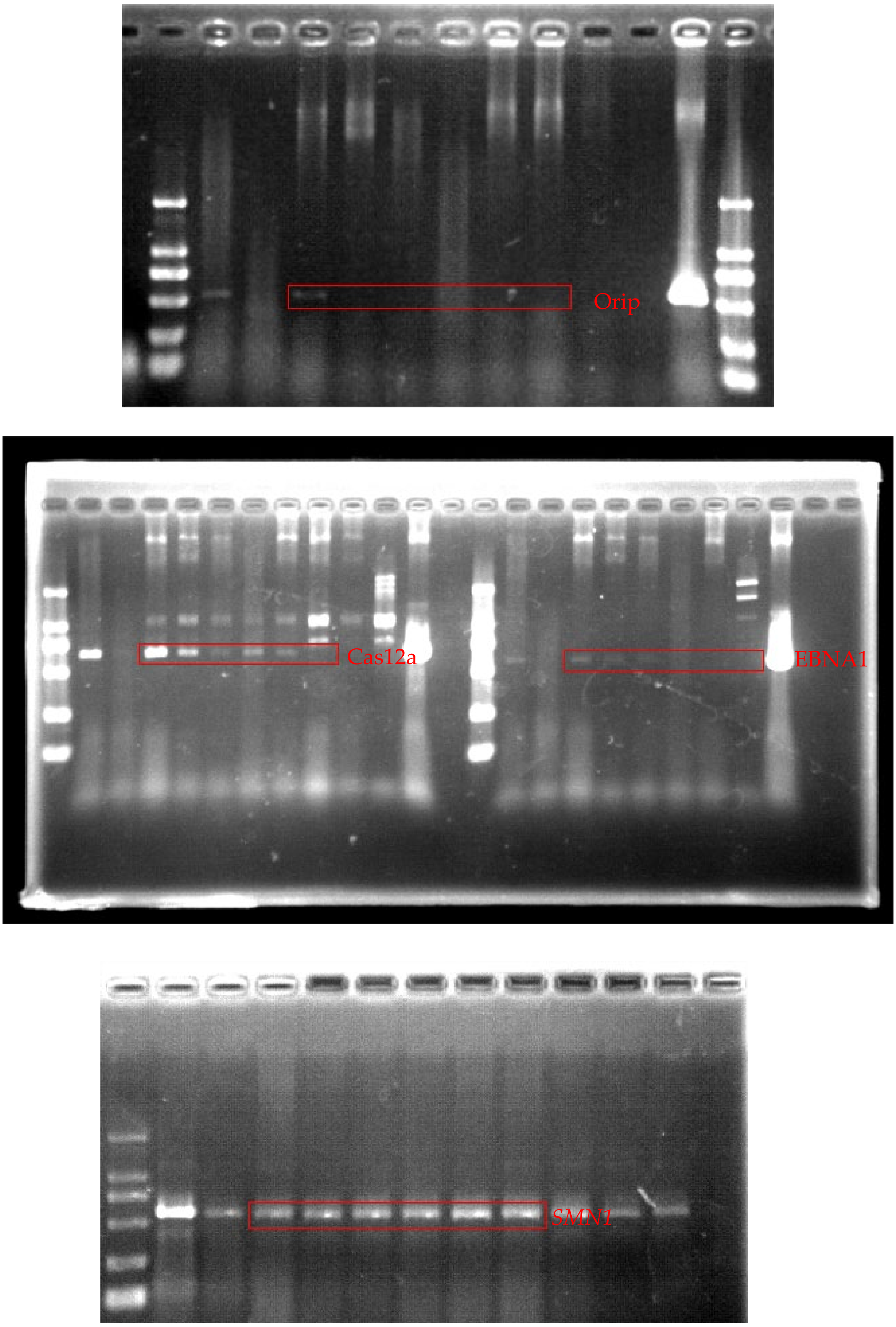

Figure S5. Agarose gel electrophoresis diagram showing all bands and molecular weight markers of Figure 3d.

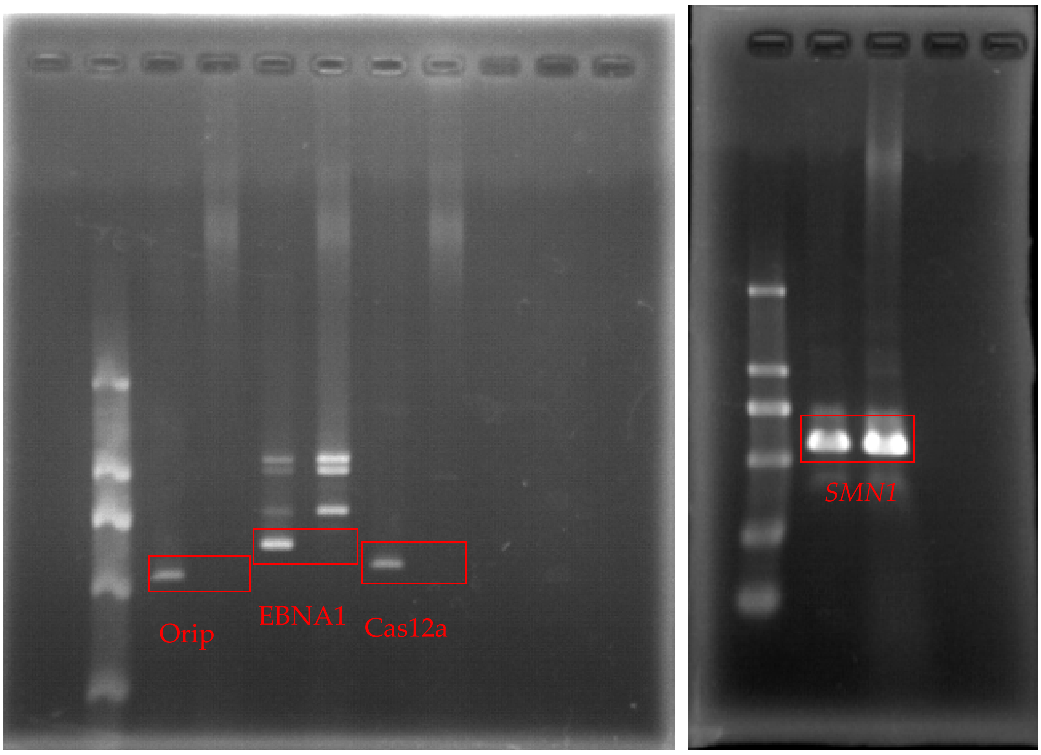

Figure S6. Agarose gel electrophoresis diagram showing all bands and molecular weight markers of Figure 3e.

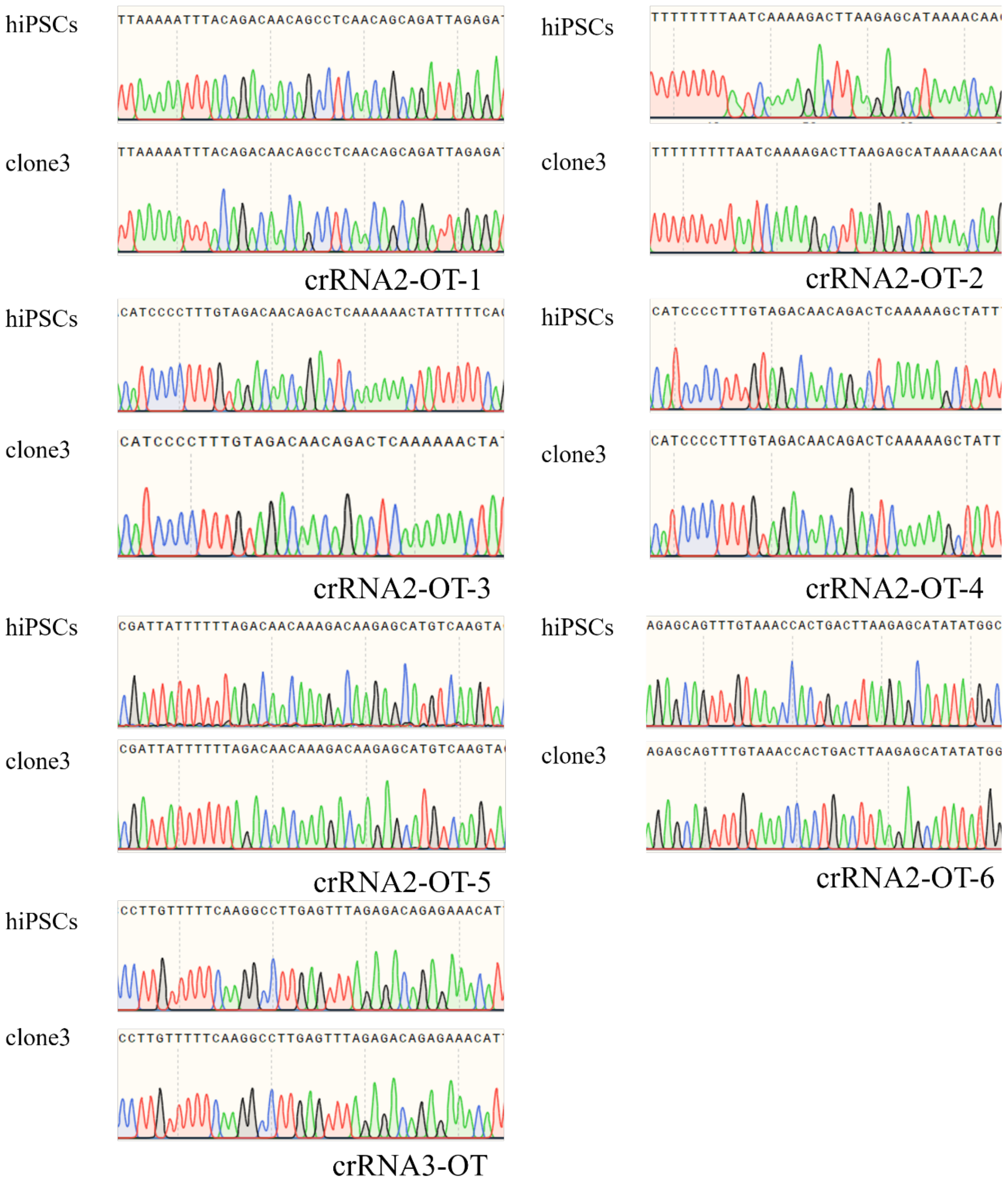

Figure S7. Sequencing results of off-target sites. Indels were detected by Sanger sequencing on seven predicted off-target sites in the positive clones. No indels was found in these sites.

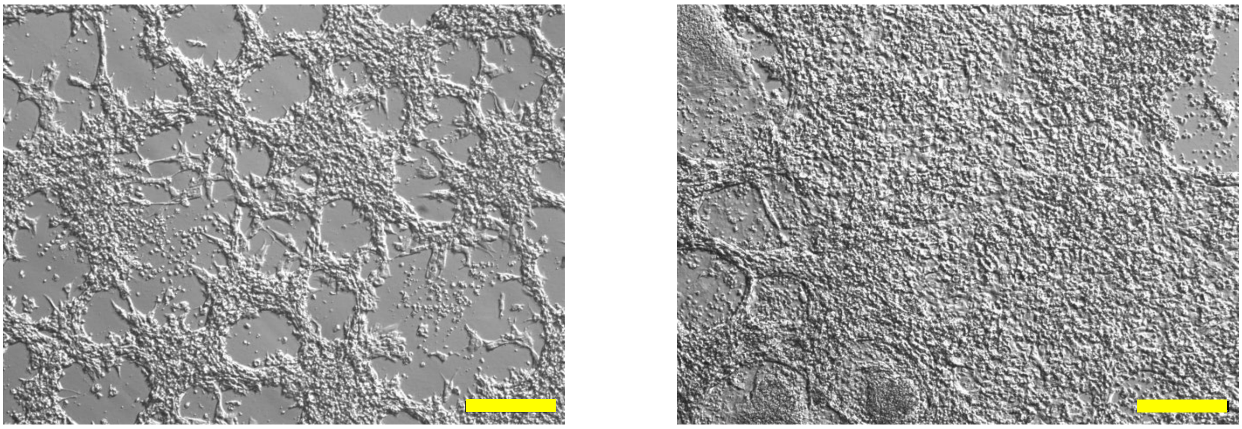

(a) (b)

Figure S8. The morphology of cardiomyocytes. (**a**) The morphology of cardiomyocytes derived from hiPSCs at the day 18. (**b**) The morphology of cardiomyocytes derived from DMD-iPSCs at the day 18. Scale bar: 250 μm.

Table S1. All the primers used in the study are showing in the Table.

Name	Sequence
Sap1	F: AGATGGAAGAGCGAGACGGGATCCCGTCTCGCTCTTCC<br>R: AAAAGGAAGAGCGAGACGGGATCCCGTCTCGCTCTTCC
U6	F: TCTATATCATAATATGAGGGCCTATTTCCCATGATTCCT<br>R: TGAGCCAATATAAATGTACaGCCGCGAATTCACTAGTGATCAATTG
GFP-crRNA	F: GATCGTCGCCGTCCAGCTCGACCAGG<br>R: AAACCTGGTCGAGCTGGACGGCGACG
NRL-crRNA	F: GATCCAAGGAAGTGCAGGCTGCAGGG<br>R: AAACCCTGCAGCCTGCACTTCCTTGG
HBB-crRNA	F: GATTGATAGGCAGCCTGCACTGGTGG<br>R: AAACCACCAGTGCAGGCTGCCTATCA
NRL	F: TTTGCAGACCTTCGCTAGTC<br>R: CAGCAGACCGCCTACATAATC
HBB	F: ATTGACCAAATCAGGGTAATTTTGC<br>R: CCAGTTTAGTAGTTGGACTTAGGG
crRNA2	F: GATTAGACAACAGACTCAAGAGCATA<br>R: AAATATGCTCTTGAGTCTGTTGTCTA
crRNA3	F: GATAAGGCCTTGAGCTTGAATACAGA<br>R: AAATCTGTATTCAAGCTCAAGGCCTT
chr2	F: AGTGTCACTCACTTCTGGAACAT<br>R: CTACGGGTGAAAGTTGCTACG
chr3	F: GGACCCACCAATGAAAGCTG<br>R: AACCTAAGCAACACTTCCTT
chr5-1	F: CAAGCAGAGCCTTTCCGGATG<br>R: GTAACACTACAAGGAACCGTCCTG
chr5-2	F: GTGCAGGCAGAGGAAAGTGGTAA<br>R: AGCCTCTAACACATTGCTTGGTTT
chr5-3	F: TGCTCCATTCTCAGCCTTGA<br>R: TTTGCCCAGCTGTAGAGGC
chr7	F: TAGCCCAACACAGGGTCTAAG<br>R: CTTTCTTGGAGCACTTACATTTGG
g3-chr2	F: GCCCTCTGGAGCCTGATTAC<br>R: ACTGCCATGGGATGTAGGC
OriP	F: TTCCACGAGGGTAGTGAACC<br>R: TCGGGGGTGTTAGAGACAAC
EBNA-1	F: ATCGTCAAAGCTGCACACAG<br>R: CCCAGGAGTCCCAGTAGTCA
Cpf1	F: TGAGACAGCCCACATCATCGC<br>R: ACCAGTCCAGCAGGTGGTACA
Del-51	F: TCTTAGAATCGTTCACTGGTTGTCC<br>R: TGAATCCTTGTTCTGCTACTTACTGG
RT-51	F: AGCAGTTCAAGCTAAACAACCGG<br>R: CCTAAGACCTGCTCAGCTTCTTC
